# Assessing insomnia management in community pharmacy setting in Jordan: A simulated patient approach

**DOI:** 10.1371/journal.pone.0226076

**Published:** 2019-12-13

**Authors:** Mayyada Wazaify, Eman Elayeh, Razan Tubeileh, Eman A. Hammad

**Affiliations:** Department of Biopharmaceutics and Clinical Pharmacy, School of Pharmacy, University of Jordan, Amman, Jordan; The University of Sydney School of Pharmacy, AUSTRALIA

## Abstract

Insomnia is one of the most common minor ailments to which patients seek advice in a community pharmacy setting. Due to the availability of a wide variety of over-the-counter (OTC) products, community pharmacists are in the front line to safe-guard patients looking for self-medication or advice for treatment of insomnia. The purpose of this study was to assess the content of community pharmacists’ interactions with patients seeking advice for insomnia in Jordan. A cross-sectional study using a simulated patient methodology was conducted across a stratified convenience sample of community pharmacies in three major cities in Jordan. The visits were evaluated using pre-defined criteria adapted from published literature relating to content and counseling skills. Visits were audio-recorded using a hidden microphone and the simulated patient completed a data collection form immediately after each visit. A total of 67 community pharmacies (response rate = 93.0%) agreed to participate and were all visited once by the simulated patient. The median duration of the visit was 2 minutes (range: 0.2–4 minutes). The majority (86.6%) of visits resulted in the sale of a drug, most commonly a combination product (paracetamol and diphenhydramine) for 30 recommendations (44.8%). This was followed by a natural plant extract combination product, namely valerian and lemon balm (*Melissa officinalis L*.) for 23 (34.3%). Pharmacists often did not question medical history or other symptoms prior to product sale. Frequently, the recommended dose (49.3%) and administration time (38.8%) were the only information provided to the patient. No information was provided in relation to potential drug interactions or contraindications. No advice was offered on lifestyle or good sleeping hygiene. This study provided evidence that community pharmacists in Jordan in general did not offer adequate counseling for patients seeking advice for insomnia. Exploration of the reasons and factors contributing to this practice and highlighting professional opportunity and responsibility is recommended.

## Introduction

Insomnia is increasingly highlighted as a common adult complaint in the modern age with estimates of up to 30% of the world population suffering from insomnia [1]. Insomnia is defined as difficulty falling asleep, staying asleep, or having restorative sleep which occurs despite an adequate opportunity for sleep and results in daytime impairment [2]. If untreated, insomnia may result in a poor quality of life, fatigue, anxiety, depression, or decreased productivity. Results from the America Insomnia Survey found that lost productivity related to insomnia contributes to a $ 63.2 billion loss a year [3]. Moreover, it is estimated that 83% of individuals who suffer from depression often experience symptoms of insomnia [2]. In the US, 150,000 road fatalities were directly linked to insomnia in 2015 [4].

Insomnia is treatable provided that optimum assessment and counseling are given to the patient. Available guidelines for the evaluation and management of insomnia in adults recommend an appropriate assessment and evaluation of the sleep complaints and advocate non-pharmacological approaches to manage sleep complaints as the first line including improved sleep hygiene and behavioral therapy techniques [2,5]. Pharmacological prescription only treatments such as short-acting benzodiazepines (e.g., temazepam) or Z-hypnotics (e.g., zopiclone) are indicated only for short term use in patients not responding to non-drug therapies [6]. Nevertheless, these are advocated with caution and after a professional consultation due to the adverse effects of their use such as next-day function deterioration, dependency, and increased risk for falls or accidents. Over-the-counter (OTC) sedating antihistamines (e.g., doxylamine and diphenhydramine) plus complementary preparations such as valerian, passionflower, hops, and melatonin are increasingly sought by patients despite the lack of evidence of efficacy and common drug-drug interactions [7].

A cross-sectional survey of 3,300 people in New South Wales, Australia revealed that over 30% of the patients with sleeping complaints resort to self-medication with alcohol and/or OTC medications or complementary preparations from community pharmacies [8]. Thus, pharmacists could pay a leading role on addressing insomnia complaints in community settings by assessing and educating patients about the use, safety, and potential precautions when using these sleep aids or advising when further medical treatment should be sought [9, 10]. Pharmacists are the most accessible healthcare professionals at the public frontline and presumably the most equipped professionals with formal knowledge and training on self-treatment and over the counter pharmacotherapy. As a result, pharmacists in community settings have a huge responsibility and opportunity to respond to patients’ minor aliment complaints and safeguard them from the risks associated with inappropriate self-medication [10]

As the rest of the world, community pharmacists in Jordan are highly accessible, and studies increasingly highlight the positive social perspectives of the public [11] and outline an increased demand to expand the role of community pharmacists [12], particularly in the supply of OTC treatments and patient counselling services [13,14].

Previous research in Jordan outlined that pharmaceutical care services are often limited and pharmacists in community settings do not collect sufficient information about patient medical history [13–17]. These studies, however, relied on self-reported questionnaires by pharmacists or patients with limited or no direct observation of the actual content and the nature of the interaction between the pharmacist and the patient in real situations.

Almaaytah et al. in 2015 used the simulated patient (SP) approach to assess the inappropriate sale of antibiotics in Jordan [16]. They highlighted the need to enforce the national pharmacy regulations prohibiting the sale of antibiotics without prescription. Also, a recent study by Hammad and colleagues in 2018 employed SP to assess OTC sales and supply and counselling practice and its content in the management of headaches [17]. They highlighted suboptimal counseling for patients seeking advice for headaches and recommended future exploration of the factors contributing to this practice. However, it is unclear whether suboptimal practice in relation to patient counselling and OTC supply is consistent across other minor aliment such as insomnia where potential risks for drug interactions and dependency is a pertinent concern that could trigger more precarious management in community settings [18,19]. Therefore, this study came to explore the content and management of insomnia complaints, to obtain insights on the potential features of future strategies needed to tackle practice gaps. The study aimed to explore community pharmacists’ management of patients seeking advice for insomnia in community settings in Jordan.

## Methods

### Study design and setting

A cross-sectional observational study using a stimulated patient (SP) approach was conducted. A simulated patient (SP), also called pseudo customer [20] or mystery shopper [21], is an individual who is trained to visit a pharmacy or other health care professional and enact a scenario previously developed by researchers to evaluate a specific behavior of the professional. This method is an advantage as it focuses on actual behavior rather than proxy measures.

There are 3,214 community pharmacies serving the community across Jordan, 56 of which are chain pharmacies incorporating around 300 branches and the remainder are privately owned independent pharmacies [22]. The study used a cluster, stratified sampling strategy based on locations in three main cities in Jordan located in the center (Amman), west (Salt), and east (Zarqa) of the country. Within each location, a convenience sample of community pharmacies was selected. A trained research assistant visited provisional pharmacies to obtain a signed consent form the pharmacist in charge. A recruitment letter was provided to all pharmacy staff working in the pharmacy explaining that a simulated patient would visit their pharmacies and seek advice to relieve the symptom of a common illness. If someone wished not to participate in the study, they were offered to wear a badge during the study period to indicate their non-participation. Details of the scenario, the simulated patient identity, and the time of the simulated visit were not revealed to the participants.

Assurance was given that any details relating to the pharmacy or the pharmacy staff would be anonymized and treated with strict confidence. Moreover, the empowerment and the voluntary nature of the project were communicated as the major aspects of the project in order to encourage pharmacists to participate. The visits were conducted within a 3-month period after consent was obtained.

To minimize detection, the research assistant who performed the simulated visits (co-author, RT) was independent from the research assistant responsible for recruitment and had not worked previously at any of the pharmacies included in the sample. The researchers (MW and EH) delivered a one-day training session focusing on enacting the scenarios in a stranded way, evaluating the visits, making an audio recording of the visit, and responding to unexpected situations during the visit. The simulated patient also consented to the ethical code designed for this study in order to maintain the anonymity of participating individuals and protect the integrity of the data obtained. Five pharmacies were visited for the study pilot and those were not included in the study analysis [18]. Visits commenced in March 2016 and extended over 12 weeks.

### Documenting the counseling process

There was only one scenario used by the SP, a female university student in her early thirties. She requested treatment for insomnia asking for something to help her to go to sleep. She did not volunteer any information unless requested. The scenario was developed and drafted by the first extensive review of the published literature and guidelines about insomnia treatment. The final version of the scenario was agreed upon through multiple meetings and discussion among the research team. The simulated patient assessed the visit using a pre-designed data collection form (Table 1). The form was completed outside the pharmacy immediately after the visit. Evaluation criteria were adopted from previously published studies [20,21,23] and modified to be applicable to pharmacy practice in Jordan. The criteria was assessed using a dichotomous scale (yes/no). The form consisted of 3 parts: (I) Assessment of content and style of information delivery, (II) Details of pharmacist recommendations, and (III) Details about the pharmacy visit including time and duration, location, business, and pharmacy staff information. The simulated patient purchased the drug that was recommended to authenticate the interaction and left the pharmacy. Data were coded and entered into SPSS© Version 20 for statistical analysis. Descriptive statistics were reported including mean ± SD, range, and median for continuous variables and frequency and percentage for categorical variables.

**Table 1:** Content of data collection form used during the simulated patient visits to community pharmacies.

**Part I: Content of Patient Counselling and Delivery Style Assessment**	
**Content of Patient Counselling** *Check if pharmacist asked about*: who had the symptoms, pattern of sleeping difficulty, duration, change in sleep environment or usual routine, trigger for sleep problem, other symptoms, actions already taken (if any), other chronic problems or medicines, counselled patient when to refer to doctor, informed patient about the time needed for medication to have an effect	**Delivery and Style Criteria** *Check if the pharmacist*: introduced him/herself, explained need for asking questions, avoided the use of inappropriate language, asked the patient if additional information (any questions) was required, checked patient preferences, offered appropriate non-pharmaceutical advice (e.g. diet, exercise, stress), offered patient access back to the pharmacy (e.g. phone number)
**Part II: Action Recommended by the Pharmacist**	
Refer to emergency department Refer to specialist doctor A pharmaceutical product *^further details below^ Other (e.g., herbal tea or no action)	If a medicine was recommended, describe below what was recommended: drug name, strength, dose, duration, etc.
**Part III: Details about the Pharmacy Visit**	
Day of the week	Duration of the visit
Time of visit	Pharmacist gender (male/ female)
Pharmacy type (Chain vs. Independent)	Pharmacist’s estimated age
Pharmacy location	Pharmacy type (Chain vs. Independent)

The audio records of all visits were independently analyzed by three investigators to validate the assessment completed by the simulated patient and minimize assessor bias. The audio-recordings were reviewed for quality assurance purposes and compared with the assessment performed by the simulated patient to minimize human error. The recordings also served to measure the duration of each visit.

### Ethics

Approval was obtained from the Institutional Review Board (IRB) at the University of Jordan Hospital (Ref number: 235/2014-30/09/2014).

## Results

### General characteristics of the sample

Of the 72 community pharmacies approached, 67 (93.0%) agreed to participate in the study. The duration of each pharmacy visit ranged between 0.20 and 5.54 minutes (median = 2.00 minutes). Table 2 summarizes the general characteristics of the participating community pharmacies. The majority of visited pharmacies were independent with no more than two customers waiting to be served. Visits were distributed equally across morning and afternoon shifts and were conducted mostly on weekdays. The majority of pharmacy staff visited (n = 38, 56.7%) were female and aged between 22–34 years.

**Table 2:** Characteristics of the community pharmacies visited by a simulated patient during insomnia study (N = 67).

Characteristics	N (%)
**Location**	
Amman	52 (77.6)
Zarka	6 (9.8)
Balqaa	9 (6.0)
**Type of Pharmacy**	
Independent Pharmacy	46 (68.7)
Chain Pharmacy	21 (31.3)
**Dispensary Load** *(i*.*e*., *number of customers at time of visit)*	
Busy (> 5 customers waiting)	0
Moderate (3–5 customers waiting)	5 (7.5)
Low (1–2 customers waiting)	18 (26.8)
Quiet (0 customers)	44 (65.7)
**Time of Visit**	
Morning Shift (9 AM– 3 PM)	34 (50.7)
Afternoon Shift (4–10 PM)	33 (49.3)
**Day of Visit**	
Weekdays	39 (58.2)
**Gender**	
Female	38 (56.7)
**Estimated Age of the Pharmacist**	
22–34 years	39 (58.2)
35–50	25 (37.3)
> 50	3 (4.4)

### Management of insomnia by community pharmacists

The pharmacists recommended a pharmaceutical product in 58 (86.6%) of the cases. The most commonly supplied drugs were a paracetamol and diphenhydramine combination (n = 30, 44.8%), followed by a valerian and lemon balm combination (n = 25, 34.3%). In three (4.5%) of the cases, prescription only medications were supplied, namely flupentixol and miletracen. In four instances, the pharmacy staff refused to offer any product or advice for insomnia. Pharmacists in a majority of cases (n = 60) did not inquire about medical history or other symptoms prior to product sale.

The majority of supplied drugs were presented to patients by their brand names (n = 49, 73.1%). In 4 cases (5.9%) generic names were provided to patients, and in the remaining 14 cases (20.8%), no names were mentioned to patients. Dose (n = 33, 49.3%) and time of administration (n = 26, 38.8%) were the two most highlighted details given to the SP. The SP was not advised about drug interactions, contraindications, or adverse reactions in any of the visits.

Regarding sleep hygiene, the SP was advised in 20 (30%) of the visits to cut caffeine intake a few hours before bedtime and in 35 (52%) visits natural products or tea extracts were recommend to be used as routine drinks around bedtime. The most commonly recommended was a drink of mixed herbs (e.g., chamomile, sage, and thyme). The other most commonly recommended drinks were anise tea (20.0%) and warm milk (15.0%) followed by valerian tea (5.0%), chamomile infusion (5.0%), and mint infusion (3.0%).

Most pharmacists used appropriate layman terms with no use of scientific jargon (98.5%). On the other hand, none of the pharmacy staff introduced him/herself to the patient, explained the need for questions, or checked the patient’s understanding or need for further information. Details of recommendations, information provided, and communication skills are presented in Tables 3 and 4.

**Table 3:** Actions recommended and information provided by community pharmacies (n = 67).

Criteria	N (%)
**Action Taken by The Pharmacist**	
Refer to emergency department	0
Refer to doctor	5 (7.5)
Supply a pharmaceutical product	58 (86.6)
No action	4 (6.0)
Recommend a natural product or herbal tea (with or without a product)	35 (52.0%)
Cut down on caffeine	20 (30.0%)
**Information Provided to Patients Who Were Supplied Drugs:**	
Brand name*	49 (84.5)
Generic name*	4 (6.9)
Dose	33 (56.9)
Form	22 (32.8)
Drug administration times of selected medication	26 (38.8)
Contraindications (if any)	0
Drug interactions (if any)	0
What must be done if the patient forgets to take the medicine	0
Side effects/ warnings	0

*In 9 cases, neither brand nor generic names were mentioned.

**Table 4:** Pharmacists’ communication skills used during the interaction with insomnia simulated patient (n = 67).

Communication Skill	Cases N (%)
1. Introduced him/herself	0
2. Explained need for asking questions	0
3. Avoided the use of inappropriate language (e.g., jargon, medical terms for a healthcare professional)*	66 (98.5)
4. Asked the patient if additional information (any questions) was required	0
5. Considered patient preferences with regard to medication choice, dosage forms, etc.	11 (16.4)
6. Checked patient understanding of recommendations	0
7. Offered patient access back to the pharmacy (e.g., phone number)	2 (3.0)

*There were one pharmacist who used the word “antihistamine” during counselling.

## Discussion

This was the second study in Jordan to use a SP to assess the content of community pharmacists’ interaction with patients seeking advice for a common minor illness and the first to evaluate OTC pharmacotherapy for insomnia. While most visits were in Amman and to independent pharmacies, visits were distributed equally between morning and afternoon shifts, weekdays versus weekends, and gender of pharmacists.

There was a high level of agreement between the different assessors regarding the scores of pharmacist-patient interactions. This could be due to the limited duration and content of interactions. The study revealed that community pharmacies in Jordan do not offer adequate counseling for patients seeking advice for insomnia. This was reported in previous research that used methods other than simulated patients [13,16,24]. However, the simulated patient approach used in this study was believed to have minimized potential bias that could have been introduced through self-report or Hawthorne effect. Thus, it has increasingly been adapted for practice feedback and professional performance evaluation [25,26].

The lack of provision of patient counselling in this study could be due to several factors such as lack of knowledge or self-esteem at the side of the pharmacist. This was concluded by Matowe et al. in a study that had been conducted in Kuwait [27]. In one study conducted in six outpatient pharmacies in a teaching hospital in Amman, the main factors believed to have contributed to lack of counselling were the high turnover of patients at the dispensary, which was not the case in our study, and the wide range of medications available at each pharmacy which made it difficult to have good command of all the information [24]. The nature of the complaint in this study could also have been a factor for not providing sufficient counselling since medications for insomnia are those that are considered liable for abuse, such as sedating antihistamines [28]. Hence, in a few cases (n = 4) pharmacists abstained from offering any help to the SP.

This study found that in most pharmacy visits (86.6%), a product was supplied but the decision and selection were done automatically and were not based on obtaining essential information about the symptoms or the patient medical history. The reported percentage in this study is less than that reported in 2010 by Kippist et al. (96.0%) from which the tool of this study was adopted [18].

Of note, a prescription only medicine (Deanxit®), which contains Flupentixol and Melitracen, was recommended over the counter in two cases. This is considered inappropriate and an illegal practice and conveys great risks to patient safety. Although this number is small, it highlights a common practice in developing countries where the regulation of drug supply and sale is not well enforced [29]. This was previously highlighted in the OTC sale of antibiotics in Jordan [16]. The study by Hammad and colleagues reported prescription only medicines including diclofenac, tinzanidine, and mefenamic acid being recommended for OTC sale in 15.8% of the SP study visits [18]. Further reports have also outlined a lack of law enforcement as a leading factor that may contribute to OTC drug abuse or misuse [28, 30,31].

The provision of information offered more often to the SP focused on drug names, doses, and time of administration. There was no emphasis on precautions or drug interactions. Neither brand nor generic names were provided to patients in almost 20.8% of the visits. This is an important finding as the ability of patients to identify medicines by name may be helpful for screening and minimizing medication errors (e.g., duplication, drug interactions or adherence problems). [31].

In Jordan, people believe in the healing power of natural products and it is a common practice to self-medicate with herbal teas [32, 33]. In Canada, Murphy and colleagues (2015) also recommended that patient counselling should move toward programs that encourage adequate consideration of nonpharmacological and alternatives [e.g., herbal drinks, good sleep hygiene; 34]. This was reflected in this study as more than half of the pharmacists recommended a natural or herbal product either with or without a medicine product. In contrast, in a study conducted in Australia by Collins et al., (2017), the supply of complementary medicines occurred in only 7% of stimulatory patient visits.[35]

### Limitations of the study

This study was mainly limited by the sample recruitment and distribution as it involved only one SP and three cities in Jordan, which might hinder generalizability to other regions in Jordan. However, similar suboptimal practice has been outlined in a previous study in the north of Jordan and a recent national survey [15, 17]. Moreover, the volunteer effect (ie- selection bias), in which pharmacists who volunteered to participate in the study were somehow different from those who did not. Such effect might also hinder generalizability to pharmacists’ population in Jordan. On the other hand, at the level of internal validity, the use of a single SP and the audit of the recordings by multiple members of the research team could be considered a strength. Also, efforts were made to minimize the Hawthorne effect and the detection of the study SP. Visits were conducted after a considerable interval (30–50 days) of the first contact and consents were obtained by a different researcher, but it was still possible that the pharmacy staff were able to detect the SP. Thus, their behavior might have been influenced. If this happened, the extent of the influence is not possible to know.

Additionally, non-verbal communication between pharmacy staff and the simulated patient was not assessed in this study. Visual recording or direct observation might help to produce objective data on these; however, this might increase risk of detection and the Hawthorne effect [36]. Furthermore, none of the pharmacy staff introduced themselves or wore identifying name tags. Thus, it was not possible to assess the association between the job title (pharmacists vs. assistants) and the management of the visits. This warrants future assessment.

## Conclusions

In summary, the assessment of insomnia-related counselling in a community pharmacy setting in Jordan displays room for improvement. There was minimal information provided to the patient regarding the basic cause (insomnia) for the visit and the delivery style of information should be improved. Future thorough research is recommended to explore the main barriers and facilitators to provide effective counselling and evidence based recommendations.

## Supporting information

S1 Data (ZIP)

S1 File (DOCX)
