# Supplementary material for: Assessing insomnia management in community pharmacy setting in Jordan: A simulated patient approach
Source: PLoS One. 2019 Dec 13;14(12):e0226076. doi: 10.1371/journal.pone.0226076 (PMC6910704; doi:10.1371/journal.pone.0226076)
Supplement: S1 Data — (ZIP) [file pone.0226076.s001.zip › simulated patient insomnia results.docx]

**Aim of the study**

This study was designed to assess practice behavior of community pharmacists in Jordan when interacting with consumers e seeking help for a self treated condition by the use of a non-prescription medicine using simulated patient methodology. From this analysis we intended to get an indication as to how prepared pharmacists in Jordan are to perform the key role expected

of them in ensuring rational use of medicines.

**Methods**

**Simulated patient methodology**

Simulated patient methodology was chosen to assess practice behavior of community pharmacists

in Jordan as it allowed for observations of pharmacists in the natural environment, not affected by awareness that their practice behavior was being predestined.

**Setting**

Cross-sectional data collection was conducted from ( ) to ( ) 2016. Investigators with the help of a research assistant recruited a convenience sample based on geographic locations from the capital city, Amman and the second largest city , Zarqa.

Upon recruitment, the research assistant explained the purpose of the study and the study methodology to prospective study participants. All pharmacists were made aware that simulated patient, would visit their pharmacies and seek advice to relieve the symptom of a common illness. To eliminate behavioral bias by knowledge about the simulated patient assessment , the simulated patient was not the same individual who took permission from pharmacists to participate in the study. Participating pharmacists were assured that data obtained would be analyzed in a confidential manner.

Anonymity, empowerment and the voluntary nature of the program were communicated as the major aspects of the project in order to encourage pharmacists to participate. For this pilot study the original target for recruitment was 20–30 pharmacies. Visits were conducted during a period of ( ) weeks.

**Scenario**

A clinical pharmacist was trained to enact the scenario: she participated in an 8-h training session,

which included both theoretical and practical aspects of enacting the simulated patient scenarios, such as the information needs of consumers of non-prescription medicines, and how to manage in situations that could emerge in the process of enacting the scenarios. Enactment of particular situations likely to emerge was practiced by role-play in order to standardize simulated patients’

responses.

The insomnia scenario in which the simulated patient was trained to enact is prescribed in (Table 1). The simulated patient would come to the participating pharmacy and ask to see the pharmacist and then seek advice for treatment of a difficulty sleeping.

The simulated patient was instructed to request treatment for difficulty sleeping and not volunteer further information unless requested by the pharmacist. The details of the scenario were only given on demand to the pharmacist. The simulated patient was instructed to communicate their request at the beginning of the encounter in a standardized way but to give no further information unless asked. If the participant asked an open-ended question, the simulated patient was to provide all relevant information but in the case of close-ended questions, she was to provide only information related to the question (most of the times ‘yes’ or ‘no’).

An expert panel encompassing three clinical pharmacists was established to review current clinical evidence in relation to the clinical scenario used in the study and to establish what represents a sensible outcome in terms of practice behavior of Jordanian community pharmacists. In response to the simulated patient’s desire for something to treat difficulty sleeping, the pharmacist should explore the patient’s history, including other conditions and medicines already tried to treat difficulty sleeping, and be able to decide whether or not the difficulty sleeping was probably to be related to stress or fatigue. The study’s expert panel agreed that the scenario warranted the recommendation of a non-prescription medicine, together with the provision of information on safe and effective use of the recommended medicine. In addition, the study’s expert panel agreed that the pharmacist should also recommend non-pharmacological treatment, such as cutting down on coffee, a warm bath 1–2 h (not immediately) before bedtime, establishing a regular bedtime and waking time, having no meals just before bedtime, no naps during the daytime, no caffeine after early afternoon, reducing extraneous noise (use earplugs if necessary) and restricting nicotine intake immediately before bedtime.

**Table 1.**

| Scenario : patient had difficulty sleeping  Simulated patient enters pharmacy and approaches dispensary. She informs the pharmacist: " I would like to have something for difficulty sleeping"  The pharmacist is given the following information, if asked:   - The drug is for the patient herself. - She is a 25-30-year-old woman who works as a teacher and studies for a master degree at night. - Due to her busy routine, she does not get enough sleep and has a poor diet. She has major exam approaching, for which she is feeling anxious and stressed - To get her schoolwork done after she gets home at night she drinks coffee. She is a non-smoker and has no recent change in her sleeping environment, attempts to sleep at a regular time, although recently she has been staying up studying late into the night. - She has not sought previous treatment - Patient has been experiencing difficulty initiating sleep or falling in sleep - She is feeling stressed and tired as a result of poor sleep - No other symptoms - No regular medications or medical conditions - If not suggested by the pharmacist, ask about the use of ‘‘natural’’ products |
| --- |

**Documenting the counseling process**

During each visit, simulated patient audio-visually recorded the interaction with the pharmacist using a hidden micro camera. Three investigators analyzed the recorded interactions.

Immediately after each visit the counseling process was documented outside the pharmacy on an

assessment form with respect to the content of the advice given including self-diagnosis, the medicine selection process, non-pharmacological recommendations , communication skills shown and any other relevant information.

Each item was evaluated according to the pharmacist’s performance in the interaction with the simulated patient measured on a dichotomic scale (yes/no).

**Results**

**General characteristics of the Community Pharmacists**

Of the ( ) community pharmacies contacted, N ( %) agreed to participate, and completed the study protocol. A total of 67 pharmacies completed the study. Pharmacies were located in the capital city of Jordan Amman and one of the biggest cities in Jordan, Zarqa.

None of the simulated pharmacist visits were excluded. None of the community pharmacies called to inform the researchers of a suspected simulated pharmacist visit during the study. The median duration of dispensation was 2 minutes , and the lowest and highest dispensation times were 0.2 and 5.54 minutes, respectively. Table 2 shows the general characteristics of the community pharmacies. 43.3% of community pharmacists were males. (Table 2 )

Overall, none of the participating pharmacist asked about other conditions that could intervene with the diagnosis or with the recommended treatment. None of the participating pharmacists checked for allergies in the simulated patient visits. (Table 3).

The most commonly recommended drugs were paracetamol and diphenhydramine (44.8%) paracetamol and diphenhydramine combination with Valerian and Melissa extracts 13.8%. In 3% of patient visits, pharmacists recommended a prescription medicine (flupentixol and miletracen).The most discussed issues, related to pharmacotherapy, were "brand name" and "dose of a drug" which were discussed in 84.5% and 56.9% of the simulated patient visits, respectively. During the counseling process, none of the pharmacists advised the simulated patient about possible drug interactions, adverse reactions or what to do if the patient forgot to take the medicine. Regarding dosage and drug administration times, these items were addressed in 32.8% 38.8 % of simulated patient visits and only two pharmacists provided appropriate non-pharmacological measure. (table 4)

Details about communication skills are listed in Table 5. Most pharmacists used appropriate terms to ensure patient comprehension (98.5%). None of the pharmacists introduced him/her self or explained the need for asking questions or checked patient understanding of recommendations.

**Table 2. general characteristics of community pharmacy/ pharmacist participating in the study. ( N= 67 pharmacies)**

| **Characteristics** | **N (%)** |
| --- | --- |
| Chain or group pharmacy | 21 (31.3) |
| Independent | 46 (68.7) |
| **Estimated dispensary load (not defined in excel)** |  |
| Busy (> 5 customers waiting) | None |
| Moderate (3-5 customers waiting) | 5 (7.5) |
| Slow (1-2 customers waiting) | 18 (26.8) |
| Quite (nil customers ) | 44 (65.7) |
| **Pharmacist gender** |  |
| Male | 29 (43.3) |
| Female | 38 (56.7) |
| **Pharmacist estimated age** |  |
| Graduate to 34 years | 38 (57.6) |
| 35-50 | 25 (37.3) |
| **>**50 | 3 (4.5) |
| **Median time of visit duration (minutes)** | 2 |

**Table 3. Frequency distribution of pharmacists’ assessment during interactions with simulated patients**

| **Did the pharmacist ask the following questions** | **Yes answers , N (%)** |
| --- | --- |
| 1. Who the medicine is for? | 12 (17.9) |
| 1. What kind of symptoms are present? | 11 (16.4) |
| 1. How long have the symptoms been present? | None |
| 1. How often do the symptoms occur? | 3 (4.5) |
| 1. What factors aggravate or relieve the symptoms ? | 6 (9.0) |
| 1. Is there other symptoms? | 2 (3.0) |
| 1. What action was taken to relieve symptoms? | 5 (7.5) |
| 1. Has the pharmacist checked for medical conditions / prescription medicines? | None |
| 1. Has the pharmacist asked about drug allergies or other allergies? | None |

**Table. 4 Frequency distribution of pharmaco-therapeutic recommendations during the interactions with simulated patient**

| **Drug selection** | **N (%)** |
| --- | --- |
| **The pharmacist indicated some drug/ product** |  |
| Refer to a specialist doctor | 5 (7.5) |
| Pharmaceutical product | 58 (86.6) |
| Nothing | 4 (6.0) |
| **Indicated drug/product** |  |
| Deanxit (flupentixol and miletracen) | 2 (3.0) |
| Valerian Officinalis Roots Extract | 4 (6.0) |
| Panadol night (paracetamol and diphynhydramine) | 30 (44.8) |
| Panadol night (paracetamol and diphynhydramine) /songha night ([Melissa Officinalis leaf extract](http://www.tabletwise.com/uae/songha-night-tablet) and Valeriana Officinalis roots extract | 8 (13.8) |
| Panadol night / chlorpheniramine | 1 (1.5) |
| Songha night | 12 (17.9) |
| Panadol night and Valerian | 1 (1.5) |
| **The pharmacist well explained the following** |  |
| Brand name | 49 (84.5) |
| Generic name | 4 (6.9) |
| Dose | 33 (56.9) |
| Form | 22 (32.8) |
| Drug administration times selected medication | 26 (38.8) |
| Contraindications (if any) | None |
| Drug interactions (if any) | None |
| What must be done if the patient forgets to take the medicine | None |
| side effects/ warning | None |
| Offered appropriate non-pharmaceutical advice | 2 (3.0) |

**Table 5. Frequency distribution of pharmacists’ communication skills used in the orientation of simulated patients**

|  | **N (%)** |
| --- | --- |
| 1. Introduced himself | None |
| 1. Explained need for asking questions | None |
| 1. Avoids the use of inappropriate language (e.g. lay terms for a healthcare professional) | 66 (98.5) |
| 1. Asked the patient if additional information ( any questions) was required | None |
| 1. Establish patient preferences | 11 (16.4) |
| 1. Checked patient understanding of recommendations | None |
| 1. Offered patient access back to the pharmacy e.g. phone number | 2 (3.0) |
