# Supplementary material for: Assessing insomnia management in community pharmacy setting in Jordan: A simulated patient approach
Source: PLoS One. 2019 Dec 13;14(12):e0226076. doi: 10.1371/journal.pone.0226076 (PMC6910704; doi:10.1371/journal.pone.0226076)
Supplement: S1 File — (DOCX) [file pone.0226076.s002.docx]

1. **صيدلية روحي- عبدون**

المريض: عندك اشي بساعد على النوم

الصيدلاني: بنادول نايت

المريض: اديه سعره؟

الصيدلاني: 2.75 دينار

المريض: ممكن فاتورة

الصيدلاني : اه طبعا ... الاسم

المريض: بتنصح باأعشاب زي ينسون

الصيدلاني: اه الينسون كتير بساعد, اشربيه و مع البنادول نايت بتنامي منيح

2مدة الزيارة: دقيقة

صبية 30

1. **صيدلية روحي - وادي صقرة**

المريض: مرحبا ... عندك اشي بساعد عالنوم

الصيدلاني: بساعد عالنوم؟ للكبار للأطفال؟

المريض: اه الي

الصيدلاني: جربتي اشي معين؟

المريض: لا

الصيدلاني: طيب جربي هذا طبيعي ماخدينه من الاعشاب (تناول سونجا نايت من الرف) ... بساعدك و ما بعمل لا تعود ولا ادمان ولا اي تأثير سلبي على الجسم

المريض: بقدر اخذ شريط فقط

الصيدلاني: لا والله بنباع باكيت فيه 30 حبة , بتستخدميه حبتين مع بعض قبل ما تنامي

المريض: بما انه اعشاب بتنصح بالاعشاب زي زهورات و ينسون

الصيدلاني: الينسون ممكن خاصة اذا عندك مشاكل بالقولون بريح , و هذا الدواء اعشاب بتقدري تستخدمي النبتة الي فيه اسمها مليسة اذا سامعة فيها بتنباع عند العطارين احكيله بدي مليسة , حتى في ناس بتزرعها حول البيت . هي ورق اخضر بتحطيه في ماء مغلي و بتشربيه.

المريض: طيب خليني اجربه شكرا

مدة الزيارة: 2 دقيقة

صبية 30

1. **صيدلية روحي – خلدا/دابوق**

المريض: مرحبا كيفك... عندك اشي بساعد على النوم

الصيدلاني:بساعد على النوم؟..أولا لمين؟

المريض: الي

الصيدلاني: هلأ ايش مشكلتك؟ مثلا جاي من بلد ثاني؟

المريض: لا صرلي هيك 7-10 ايام بلاقي صعوبة اني اغفا او ابلش انام فبطول لأغفا و بضل طول نهار تعبانو فبدي اشي يخليني انام

الصيدلاني: هلأ نحنا حنبدا في الأشيا الطبيعية ... بدك تبدي انتي خدي بابونج و ينسون بليل قبل ما تنامي بساعد كتير, و اعملي رلاكس, و كمان افحصي الحديد لانه ممكن انه ما بساعد انك تستغرقي بالنوم... خليني اشوف عينيكي

المريض: اوكي

الصيدلاني: و اذا ما استفدتي ممكن اعطيكي بنادوا نايت ... خلينا نبلش بهاي الاشيا و بعدين منشوف هو باراسيتامول مع انتي هيستامين هو safe

المريض: خليني اشوف و برجعلك شكرا

مدة الزيارة: 2.3 دقيقة

انثى 30

1. **صيدلية التلال – تلاع العلي**

المريض: مرحبا ... عندك اشي بساعد على النوم

الصيدلاني: مممم... الك؟

المريض:اه

الصيدلاني: هذا اعشاب هاي العشبة بتنيم بتاخدي حبتين قبل النوم ب 15 دقيقة (نيرفيكان)

المريض: اديه سعره

الصيدلاني: 10.48 دينار

المريض: طيب شو رأيك بالزهورات و الينسون الأعشاب بشكل عام ؟

الصيدلاني:هلا حسب الاجسام في ناس بناموا و بستفيدوا و في ناس لا... انتي في قلق يعني؟

المريض: لا ما في شي بس صرلي تقريبا اسبوع مو عارفة أغفى فطول الوقت تعبانة فبدي اشي

الصيدلاني: يخليكي تنامي؟

المريض: اه

الصيدلاني: طيب هذا ما بخوف (مشيرة الى الدواء) لانه اعشاب ...

المريض: لم اجلب فلوس كفاية برجعلك

الصيدلاني: طيب

المريض: بس مستغليتها صراحة و حابة اجرب الزهورات و الينسون

الصيدلاني: براحتك صراحة ... بس هاي مو ينسون هاي نبتة اسمها فاليريانا روت اقرأي عنها

مدة الزيارة 3 دقيقة

صبية في اواخر 20

1. **صيدلية خالدة – تلاع العلي**

المريض: في عندك شي بساعد عالنوم

الصيدلاني:بساعد عالنوم؟ لا والله في سونجا نايت اذي بدك

المريض: اه نصحوني في الناس

الصيدلاني: بتنصحيني بالاعشاب (زهورات ينسون؟)

الينسون ممكن بساعد... ما بدك هذا (مشيرة لسونجا نايت)

مدة الزيارة: 1 دقيقة

انثى 30

1. **صيدلية خميس - شارع عكا جبل الحسين مسا 9**

المريض: السلام عليكم ... عندك اشي بساعد عالنوم

الصيدلاني: فش غير البنادول نايت

المريض:طيب شو بتنصح بالنسبة للأعشاب ينسون زهورات

الصيدلاني: اه خلطة ينسون زهورات منيح

المريض: طيب شكرا

1. **صيدلية الفخر – جبل الحسين**

المريض: السلام عليكم ... في عندك اشي بساعد عالنوم

الصيدلاني:اه فيه بنادول نايت او في سونجا نايت هذا اعشاب

المريض: في اشيا اكتر دواء

الصيدلاني: اي شي تاني بده استشارة دكتور عشان يشخص الحالة بالضبط و يكون معاكي وصفة

مدة الزيارة: 1 دقيقة

شاب في 30

1. **صيدلية شوكت – جبل الحسين**

المريض: مرحبا ... عندك اشي بساعد عالنوم

الصيدلاني: في بنادول نايت يا عمي بس

المريض:طيب بتنصح بالعشاب

الصيدلاني: اه الينسون

المريض:شكرا

الصيدلاني: عفوا

مدة الزيارة: 1 دقيقة

رجل 40

1. **صيدلية خالد بن الوليد – جبل الحسين المسا 8**

المريض: مرحبا .. في عندك اشي بساعد على النوم

الصيدلاني:خدي بنادول نايت (لم تعير الموضوع اي اهمية و ادارت ظهرها)

المريض:عندي منه...طيب بالنسبة للاعشاب الينسون الزهورات

الصيدلاني: الينسون بهدي كتير منيح

المريض: طيب شكرا

المدة 1 دقيقة

صبية لآخر 20

1. **صيدلية بلازما فارما – جبل الحسين المسا 8**

المريض: مرحبا ... (في زائر) في عندك اشي بساعد عالنوم

الصيدلاني: في بنادول نايت او سونجا نايت بس

المريض:طيب شو بالنسبة للاعشاب زي ينسون و زهورات

الصيدلاني: اشربي لبن او لبن مخيض بساعد عالنوم (افتراض انه الي)

المريض: طيب شكرا

مدة الزيارة 1 دقثقة

رجل 50

1. **صيدلية أحمد – مقابل الجامعة صويلح المسا 6**

المريض: في زائر 2 ...مرحبا عندك اشي بساعد على النوم؟

الصيدلاني: في عندك البنادول نايت و السونجا نايت بس.. السونجا نايت بتميز انه طبيعي فيه مادة الفليريانا الي بتساعدك على النوم هاي مش مادة كيماوية اذا بتحبي اشي طبيعي ... طبعا في اشيا اقوى بس ما منجيبها الا عالطلب بتكون لازم بوصفة استشاري

الكريض: طيب شو بالنسبة للينسون و الزهورات

الصيدلاني: الينسون بنعس يعني بساعد... انتي من النوع الي بنعس بسرعة؟

المريض: لا انا جديد صرلي 7 ايام انه مو عارفة اغفا

الصيدلاني: عندك احتقان حاليا عشان اقلك تضربي عصفورين بحجر ... اعراض رشح

المريض: لا ما ربطها بشي صراحة

الصيدلاني: لانه في ناس بياخدو بنادول كولد اند فلو بياخدو حبتين قبل النوم بنعس كتير بسطل

المريض: عندي منه بالبيت

طيب اوكي شكرا

مدة الزيارة 2ز5 دقيقة

صبية 25

1. **صيدلية ريميديز – شارع مكة صبح 1**

المريض: مرحبا ... عندك اشي بساعد عالنوم

الصيدلاني: اه في مثلا بنادول نايت اذا مجربتيه و في مثلا زي اعشاب (سونجا نايت و فاليريان) عادة حبتين قبل النوم هم عبارة عن مليسة

المريض: شو احسن

الصيدلاني: السونجا نايت احسن لانه تركيزها احسن

المريض: اه

الصيدلاني: كخبرة من الناس اكثر شي بستفيدو عليه السونجا...و نحنا عنا كمان الميلاتونين هو هرمون بفرزه الجسم عشان تقدري تنامي هو منوصي عليه من برا بس حاليا موجود... مفيد للناس الي بتسافر و بتخربط ساعات نومهم ممكن نتيجة jet lag او فرق توقيت و بتاخد عاسبوعين 3 و بعدين خلص بتبطلي تحتاجيه

المريض: طيب خليني افكر اكثر

الصيدلاني : طيب اوكي

المدة: 3.5 دقيقة

صبيتين اول 30

1. **صيدلية صيام – صويلح**

المريض: (زوار1) مرحبا عندك اشي بتساعد عالنوم

الصيدلاني: لمين

المريض: الي

الصيدلاني: ليش ما بتنامي

المريض: ما بعرف ما ربطها بشي صرلي 7-10 ايام هيك

الصيدلاني: بدك وصفة هذا بنزل ضغطك و بعملك مشاكل

المريض: لازم وصفة ..اه .. طيب شو بالنسبة للأعشاب

الصيدلاني: الينسون بهدي و ممكن تاخدي دوا حساسية

المريض: طيب شكرا

المدة: 1 دقيقة

صيدلانية 25

صيدلاني 50

1. **صيدلية دواكم – صويلح**

المريض: مرحبا في عندك اشي بساعد على النوم

الصيدلاني: لمين

المريض:الي

الصيدلاني:الك انتي ليش ما بتنامي

المريض: صرلي 7-10 ايام هيك

الصيدلاني: التوتر و التفكير كلها عوامل بتلعب دور جربي البنادول نايت

المريض: عندي زيه

الصيدلاني: طيب في سونجا نايت زي تركيبة نبتة بتساعد على النوم

المريض: طيب بالنسبة للاعشاب

الصيدلاني : اه الينبون و حليب فاتر كتير بساعد

المريض: اوكي شكرا

الصيدلاني: في مهدئات بس لازمها وصفة طبية بعدين بتتعودي عليها بصراحة

المريض: اه

الصيدلي: كل شي اله اثار جانبية اذا الواحد بقدر يحل القصة خلص بحلها

المريض: طيب شكرا

المدة:1.45 دقيقة

صبية 30

1. **صيدلية الباكالوريا - الفحيص**

المريض : مرحبا في عندك اشي بساعد عالنوم ؟

الصيدلاني: اشي بساعد على النوم؟ ان شاء الله...بتاخدي منه حبتين هذا دوا طبيعي (مشير للسونجا)

المريض: اه عندي زيه

الصيدلاني:اي شي ثاني بحتاج وصفة

المريض: اه طيب شو بالنسبة للاعشاب زي الينسون و الزهورات

الصيدلاني: اه يعني ممكن تساعدك بس الاحسن تاخديهم مع السونجا نايت قبل النوم بساعة

مدة الزيارة: 1.3 دقيقة

رجل 40

1. **صيدلية ساندرا – الفحيص**

المريض : مرحبا في عندك اشي بساعد عالنوم ؟

الصيدلاني: اشي بساعد عالنوم.؟؟؟ قصدك مهدئ؟ ممكن اعطيكي اعشاب

المريض: زي ايش؟

الصيدلاني: السونجا نايت ...انتي مجربة اشي من قبل ... هو بالاول الك اصلا؟

المريض: لا مو مجربة و اه الي

الصيدلاني: الك..طيب قلق؟ وجع؟

المريض: ما ربطه بشي لا بس صرلي 7-10 ايام بلاقي صعوبة اغفى فبضل طول اليوم تعبانة

الصيدلاني: اكيد هلا المخ شغال عرفتي يعني مش انه تعب او وجع في الجسم

المريض: لا لا

الصيدلاني: بقدر اساعدك فيه انه اعطيكي بس السونجا نايت هي اعشاب بتساعد على النوم

المريض: اديه سعره

الصيدلاني: 7 دينار

المريض: ممكن فاتورة (دخول زائر)

الصيدلاني : اه طبعا .. اسمك؟

المدة: 2.5 دقيقة

1. **صيدلية عاشور - السلط**

المريض : مرحبا في عندك اشي بساعد عالنوم ؟

الصيدلاني: بس بوصفة

المريض:طيب الاعشاب ممكن تساعد

الصيدلاني: الاعشاب بتساعد يعني مثلا لو تاخدي بابونج نعنع و شغلات زي هيك

المريض: طيب شكرا

مدة الزيارة: 45 ثانية

رجل 50

1. **صيدلية ميسرة - السلط صباحا 11**

المريض : زوار 2...مرحبا في عندك اشي بساعد عالنوم ؟

الصيدلاني: بنادول نايت او سونجا اي واحد بدك؟

المريض: طيب اشوف التاني

الصيدلاني: السونجا؟...

المريض: اه ممكن فاتورة

الصيدلاني : الاسم

المريض: شو بالنسبة للأعشاب

الصيدلاني: الينسون

(الزوار صوتهم عالي )

مدة 3 دقيقة

صبية 35

1. **صيدلية الشرق - الزرقاء**

المريض : مرحبا في عندك اشي بساعد عالنوم ؟ (زوار 2)

الصيدلاني: اشي بساعد عالنوم؟

المريض: اه

الصيدلاني:في البنادول نايت بساعدك عالنوم (الزوار يتدخلو و ينصحوا باللبن و اليانسون و يشجعوا البنادول نايت و الصيدلانية وافقتهم )

المريض:اشوفه .. اه عندي زيه شكرا

الصيدلاني: انا ما بحب اعطي مهدئات بس ممكن تاخدي مع البنادول نايت حبة الليرفين او فيناليرج اي دوا من تاعون الحساسية ممكن اعطيكي حبة تريحك و تساعدك عالنوم خديها حبة قبل النوم مع حبة بنادول نايت .

المريض: ممكن فاتولرة

الصيدلاني: الاسم؟...

المريض: طيب شكرا

الصيدلاني: لا تشربي منبهات في الليل و خذي فايتمين سي بتريح الاعصاب... الف سلامة عليكي و طمنينا عنك

المريض: شكرا

مدة الزيارة : 9.37 ...بس موضوعي 3 دقثقة

صبية 36

1. **صيدلية جوهرة مكة - الزرقاء**

المريض : مرحبا في عندك اشي بساعد عالنوم ؟

الصيدلاني: بساعد عالنوم ...لا والله حتى بنادول نايت خالص (افترض انه انا)

المريض: شكرا

المدة: 35 ثانية

الصيدلاني رجل 45

1. **صيدلية عمر الحديثة - الزرقاء**

المريض : مرحبا في عندك اشي بساعد عالنوم ؟

الصيدلاني: منوم يعني حاليا لا... لمين بدك اياه؟

المريض:الي

الصيدلاني:ليه

المريض:صرلي 7-10 ايام مو عارفة انام

الصيدلي: حاليا ما في بس بكرة بدبرلك اكمن حبة يمشو حالك (10 حبات)

المريض: شكرا .. بس ممكن اسال اذا بتنصح بالاعشاب زي الينسون و الزهورات (بالغلط طفا جهز التسجيل)

الصيدلي: اه ممكن خلطة تفيد

المريض: شكرا

المدة 1.20 دقيقة

رجل 55

1. **صيدلية بشرى - الزرقاء**

المريض : مرحبا في عندك اشي بساعد عالنوم ؟

الصيدلاني: لا والله بس بنادول نايتشو بدك تنامي يعني ...بنادول نايت بخليكي تدخلي في مود النوم (افتراض انه الي)

المريض: اه اشوف شكله بلكي عندي زيه

الصيدلاني:منوم منوم ما بصير نبيع الا بوصفة

المريض: اه طيب .. بتنصح بالاعشاب

الصيدلاني: اشربي حليب

المريض: شكرا

المدة 1 دقيقة

صبية 35

1. **صيدلية الأسرة - الزرقاء**

المريض : مرحبا في عندك اشي بساعد عالنوم ؟

الصيدلاني: اشي بساعد عالنوم ... في بنادول نايت

المريض:طيب ...اديه سعره

الصيدلاني: حبتين قبل النوم 2.75

المريض:معلش فاتورة ..بس بتنصح بالاعشاب

الصيدلاني: اه طبعا ممكن تفيد

المريض: شكرا

المدة: 1.4 دقثقة

شاب 33

1. **صيدلية توفيق - الزرقاء الساعة 4**

المريض : مرحبا في عندك اشي بساعد عالنوم ؟ (زوار 1)

الصيدلاني: بساعد عالنوم في

المريض:ايش عندك

الصيدلاني: تناول من الرف ...هذه حبوب فالريان حبوب طبيعي بتاخذ حبتين 3 مرات باليوم بهدأ الشخص و بساعد عالنوم

المريض: اديه سعره

الصيدلاني: سعره 6.30 دينار

المريض: معلش فاتورة؟

الصيدلاني: اه طبعا تفضلي

المريض: بتنصح بالاعشاب زي الزهورات

الصيدلاني: اه الينسون بهدي كمان (من بعيد بصوت خافت) و اشربي كاسة حليب قبل النوم بتهدي.. الف سلامة

المريض: شكرا

المدة 3.5 دقيقة

رجل 40

1. **صيدلية فلسطين الحديثة - الزرقاء**

المريض : مرحبا في عندك اشي بساعد عالنوم ؟

الصيدلاني: لا ما عنا شوفي دكتور

المريض: طيب شكرا

المدة 20 ثانية

صبية 28

بالغلط لم اسجل
